# Pelvic radiotherapy for cervical cancer affects importantly the reproducibility of cytological alterations evaluation

**DOI:** 10.1186/s12907-018-0078-z

**Published:** 2018-10-05

**Authors:** Fernanda A. Lucena, Ricardo F. A. Costa, Maira D. Stein, Carlos E. M. C. Andrade, Geórgia F. Cintra, Marcelo A. Vieira, Rozany M. Dufloth, José Humberto T. G. Fregnani, Ricardo dos Reis

**Affiliations:** 1 Faculty of Health Science of Barretos Dr. Paulo Prata, Avenida Loja Maçônica Renovadora 68, Nº 100, Barretos, 14785-002 São Paulo Brazil; 2 Department of Pathology, Barretos Cancer Hospital, Rua Antenor Duarte Villela, 1331 - Dr. Paulo Prata, Barretos, 14784-400 São Paulo Brazil; 3 Department of Gynecologic Oncology, Barretos Cancer Hospital, Rua Antenor Duarte Villela, 1331 - Dr. Paulo Prata, Barretos, 14784-400 São Paulo Brazil; 4 Post-Graduation Program in Oncology, Barretos Cancer Hospital, Rua Antenor Duarte Villela, 1331 - Dr. Paulo Prata, Barretos, 14784-400 São Paulo Brazil

**Keywords:** Cervical cytology, Pelvic radiotherapy, Reproducibility, Cervical cancer, Post radiotherapy cells changes

## Abstract

**Background:**

to evaluate the intraobserver and interobserver reproducibility of cervical cytopathology according to previous knowledge of whether patients received radiotherapy (RT) treatment or not.

**Methods:**

The study analyzed a sample of 95 cervix cytological slides; 24 with cytological abnormalities (CA) and presence of RT; 21 without CA and presence of RT; 25 without CA and without previous RT; 25 with CA and without previous RT. Two cytopathology (CP) evaluations of the slides were carried out. For the first CP re-evaluation, the cytotechnologist was blinded for the information of previous RT. For the second CP re-evaluation, the cytotechnologist was informed about previous RT. The results were analyzed through inter and intraobserver agreement using the unweighted and weighted kappa.

**Results:**

Post radiotherapy effects were identified in 44.4% of cases that undergone previous pelvic RT. The agreement for RT status was 66.32% (unweighted K = 0.31, 95%CI: 0.13; 0.49, moderate agreement). The intraobserver agreement, regarding the cytological diagnoses, regardless of radiotherapy status, was 80.32% (weighted K = 0.52, 95%CI: 0.34; 0.68). In no RT group, the intraobserver agreement was 70% (weighted K = 0.47, 95%CI: 0.27;0.65) and in patients that received RT, the intraobserver agreement was 84.09% (unweighted K = 0.37, 95%CI: 0.01;0.74). The interobserver agreement between cytopathology result (abnormal or normal) in the group with RT, considering normal and abnormal CP diagnosis was 14.0% and 12.5%, respectively. There was no association between the cytological alterations and the median time between the end of RT and the cytological diagnosis.

**Conclusion:**

This study showed that RT has an important impact in CP diagnosis because the agreement, also in interobserver and intraobserver analysis, had high discrepancy in patients that received RT. Also, demonstrated that it is difficult to recognize the presence of RT in cytological slides when this information is not provided.

**Electronic supplementary material:**

The online version of this article (10.1186/s12907-018-0078-z) contains supplementary material, which is available to authorized users.

## Background

Radiotherapy (RT) is a necessary and frequently used procedure for the treatment of gynecological cancer [1]. However, RT may have adverse effects on the lower genital tract after maximal therapeutic doses. Squamous cells undergo major changes due to the effect of RT on the vagina and cervix, often leading to difficulties to obtain proper diagnosis [2]. The Pap test is a useful tool for screening of precursor lesions detection prior to progression to invasive carcinoma forms, where the results are described through the Bethesda system [3, 4]. In contrast, cytology has limited sensitivity for detection of residual cervical cancer after radiotherapy [5, 6].

Some authors suggest that patients with a history of previous RT have Pap tests indicating an increase in the incidence of unsatisfactory sample slides, from 4.3 to 13.2% when evaluated using satisfactory cellularity criteria (> 8000 squamous cells and < 75% of the obscure epithelium) [7]. In addition, the study by Shield et al. showed cytology presenting marked effects of RT in 29.2% of cases [2].

Some studies show that it is important to properly identify the types of abnormal cells present in smears under the influence of RT. This leads to determine how many of these modifications are due to a possible recurrence of the tumor or to a new neoplastic lesion [2, 8]. Actually, follow-up of patients treated for cervical cancer based on routine Pap smears does not permit earlier detection of recurrence and does not increase survival [9].

The literature is scarce regarding information on intraobserver performance and reproducibility in cytological exams after radiotherapy. On the other hand, its known that cytology has limited accuracy also in women not treated by radiotherapy [10, 11].It is observed the existence of a low interobserver agreement in the sample of patients who received RT, which is expected due to the bias induced by radiation, thus favoring the cytological categorization of uncertainty [12].

The objective of this study was to evaluate the intraobserver and interobserver reproducibility of cervical cytology exams according to the previous knowledge of whether the patient received RT or not.

## Methods

The study was approved by the Research Ethics Committee of Barretos Cancer Hospital (BCH, Brazil) and designed to analyze intraobserver reproducibility of Pap smear result. The sample consisted initially of 100 slides of normal and abnormal cytological exams from patients treated at BCH in the Gynecology Oncology outpatient clinic and in the Prevention Department. This work did not include live subjects and also it was not necessary informed consent due to the design of the study. Demographic and diagnosis data (original cytopathology evaluation), type of CA and treatment length were collected from medical chart.

The samples were selected from a bank of slides – prepared using the method of liquid-based cytology in order to proceed to the cytological examination – and collected in 2010 and 2011. A sampling method stratified by Pap result (normal and abnormal) and radiotherapy status was adopted in order to select the slides.

**Fig. 1:**
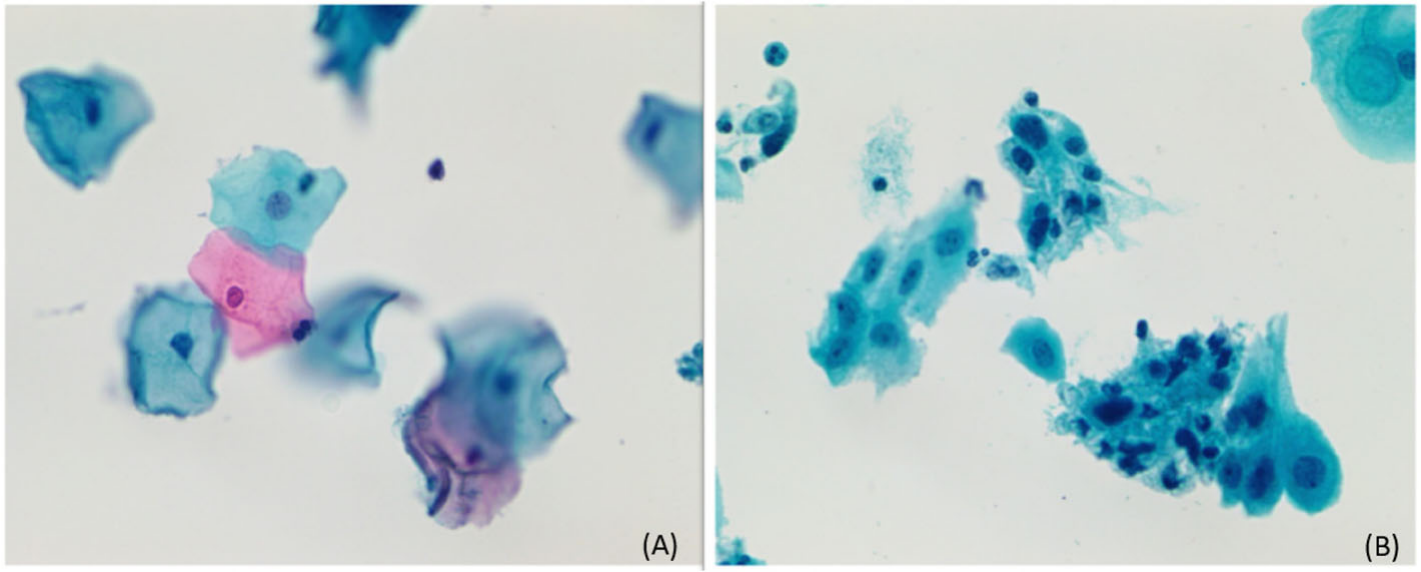
**a** Cytological Findings: Normal Squamous cells by Papanicolaou stain. **b** Radiotherapy changes. The sheet shows nuclear enlargement and altered cytoplasmic staining by Papanicolaou stain

Five slides were excluded from the study: one slide that had not been prepared on liquid-based cytology, and four slides that the cytotechnologist considered inadequate to perform the review. Therefore, the final sample consisted of 95 slides, 50 slides of cytological exams with abnormal (25) and normal (25) results in patients without previous pelvic RT and 45 slides of cytological exams with abnormal (24) and normal (21) results in patients who received previous pelvic RT for cervical cancer treatment. All patients received concomitant radiochemotherapy and the radiotherapy protocol was EBRT and Brachytherapy. The cytological changes were classified according to Bethesda system [4]. The Fig. 1 shows the difference between normal cytological findings: (**a**) Normal Squamous cells and (**b**) Radiotherapy changes: Nuclear enlargement and altered cytoplasmic staining. Both by Papanicolaou stain.

A senior cytotechnologist and a senior gynecologic oncology pathologist conducted the cytopathology (CP) re-evaluations, following the routine of the pathology department at BCH. The department’s pathologist re-evaluated all slides that the cytotechnologist considered abnormal. In the first CP re-evaluation, 95 slides were evaluated with no information regarding the result of the original CP evaluation of the cytological exam (gold standard in this study) and the presence or absence of previous RT. Besides performing the CP evaluation, slides containing cells with signs of post radiotherapy effects were identified. At the second CP re-evaluation, conducted 6 months later, the same observers re-evaluated the 95 slides having no information on the result of the original diagnosis of the cytological exam, but knowing whether the patient received previous pelvic RT.

The agreement for RT status was calculated using unweighted kappa. For CP evaluation, three agreement analyses were conducted: considering all patients, patients with no RT and patients with RT. In the first analysis, the agreement (K^1^) between the original CP evaluation and the first CP re-evaluation (blinded for RT information and cytological result) was evaluated. In the second analysis, the agreement (K^2^) between the original CP evaluation and the second CP re-evaluation (blinded only for the cytological result, but not for RT status) was evaluated. K^1^ and K^2^ were considered interobserver assessment. In the third analysis, the intraobserver agreement (K^3^) between the cytological result of the first CP re-evaluation and the second CP re-evaluation was evaluated. Weighted kappa was used to evaluate the agreement between the three evaluation phases; concordant results were given full credit [1], discordant results off by a single category were given half credit (0.5) and discordant results off by more than one category were given no credit (0). The values and the order of cytological results can be observed in Table 1. The agreement between the original CP evaluation and the first CP re-evaluation as well as between the second re-evaluation and the first re-evaluation considering the cytopathological diagnosis (normal and abnormal) and RT status (absence and presence), was evaluated using the unweighted kappa.

**Table 1:** Weight scheme for agreement analysis

	Negative	ASC-US	LSIL	AGC	ASC-H	HSIL	ADENO/CEC
Negative	1						
ASC-US	0.5	1					
LSIL	0	0.5	1				
AGC	0	0	0.5	1			
ASC-H	0	0	0	0.5	1		
HSIL	0	0	0	0	0.5	1	
ADENO/CEC	0	0	0	0	0	0.5	1

*ADENO/CEC* Adenocarcinoma, *AGC* Atypical Glandular cells, *ASC-H* Atypical Squamous Cells cannot exclude HSIL, *ASC-US* Atypical Squamous Cells of Undetermined Significance, *HSIL* High Grade Squamous Intraepithelial lesion, *LSIL* Low Grade Squamous Intraepithelial lesion

According to Landis and Koch [13], Kappa index is interpreted as follows: a range of 0.00–0.20 indicates slight agreement, a range of 0.21–0.40 indicates reasonable agreement, a range of 0.41–0.60 indicates moderate agreement, a range of 0.61–0.80 indicates substantial agreement, a range higher than 0.80 indicates near perfect agreement and 1.0 indicates perfect agreement. Chi-square test was used to evaluate the frequency and association of cytological abnormalities according to the time elapsed after RT and age. All analyzes were performed using Stata/MP 14.1 software. The level of statistical significance was set at 5%.

## Results

In this study, 95 liquid-based cytology slides were analyzed. The median age was 51 years (17–91 years). Patients with abnormal cytology (49 individuals) were divided according to their age (below and above 50 years old). Of this group with abnormal cytology just one had histologically confirmed CIN2. Using Chi-square test, it was possible to verify that there was no association between age and types of CA (*p* = 0.095).

Of the 95 patients evaluated, 45 had undergone previous pelvic RT. In the first CP re-evaluation, post radiotherapy signs were identified in the cells of 27 slides (28.4%), of which 20 slides were from patients that actually undergone RT (74.1%). Therefore, the cytotechnologist was able to detect post radiotherapy effects in 44.4% (20/45) of cases that undergone previous pelvic RT. The agreement for RT status was 66.32% (unweighted K = 0.31, 95%CI: 0.13–0.49, moderate agreement).

Regarding the CP evaluations (original, 1st and 2nd re-evaluation), results are depicted in Table 2. The diagnoses with highest frequencies were: negative (48.4% in the original evaluation; 70.5% in the 1st re-evaluation and 57.4% in the 2nd re-evaluation), ASC-US (13.7% in the 1st re-evaluation; 26.6% in the 2nd re-evaluation) and ASC-H (18.9% in the original evaluation).

**Table 2:** Results of analysis of slides, distributed by number and frequency

	Negative	ASC-US	LSIL	AGC	ASC-H	HSIL	ADENO/CEC	Total
Original evaluation	46 48.4%	9 9.5%	7 7.4%	3 3.2%	18 18.9%	9 9.5%	3 3.2%	95 100%
1st Re-evaluation	67 70.5%	13 13.7%	5 5.3%	1 1.1%	6 6.3%	3 3.2%	0 0%	95 100%
2nd Re-evaluation	54 57.4%	25 26.6%	3 3.2%	2 2.1%	6 6.4%	4 4.3%	0 0%	94 100%

*ADENO/CEC* Adenocarcinoma, *AGC* Atypical Glandular cells, *ASC-H* Atypical Squamous Cells cannot exclude HSIL, *ASC-US* Atypical Squamous Cells of Undetermined Significance, *HSIL* High Grade Squamous Intraepithelial lesion, *LSIL* Low Grade Squamous Intraepithelial lesion

Table 3 shows the results of agreement values regarding the cytological diagnoses, considering all cases and RT status. Considering all patients, regardless of radiotherapy status, the intraobserver agreement was 80.32% (weighted K = 0.52, 95%CI: 0.34–0.68). In the patients that did not undergone RT, the intraobserver agreement was 70% (weighted K = 0.47, 95%CI: 0.27–0.65). In patients that undergone RT, the intraobserver agreement was 84.09% (unweighted K = 0.37, 95%CI: 0.01–0.74). An additional file with Additional file 1: Tables S1 to S9 show the values for the cytological diagnoses according to RT Status.

**Table 3:** Values of agreements between cytopathological diagnosis

Patients	Evaluation	Exact Agreement	Expected Agreement	Weighted Kappa	95% Confidence Interval	N
	K^1^	49.47%	45.74%	0.069	−0.05; 0.18	95
All	K^2^	47.87%	44.11%	0.067	−0.05; 0.19	94
	K^3^	80.32%	59.40%	0.515	0.34; 0.68	94
	K^1^	51.00%	42.44%	0.149	−0.02;0.32	50
No	K^2^	47.00%	39.78%	0.120	−0.04;0.31	50
RT	K^3^	70.00%	43.18%	0.472	0.27;0.65	50
	K^1^	47.78%	46.91%	0.016	−0,03;0.06	45
RT	K^2^	48.86%	46.69%	0.041	−0.01;0.09	44
	K^3^	84.09%	74.59%	0.374^a^	0.01;0.74	44

^a^Unweighted kappa, Less than three order categories

K^1^: original vs first re-evaluation; K^2^: original vs second re-evaluation; K^3^: first re-evaluation vs second re-evaluation; *RT* radiotherapy

Table 4 demonstrates the agreement between cytopathology result (abnormal or normal) and post radiotherapy effect on the cells of the smear identified during 1st re-evaluation. Considering original normal CP diagnosis with RT, of 21 patients, there was agreement in 3 (14.0%) patients, while, considering original abnormal CP diagnosis with RT, of 24 patients, there was agreement in 3 (12.5%) patients. On the other hand, in 2nd evaluation normal CP diagnosis with RT, of 36 patients, there was agreement in 13 (36.0%) patients, while in 2nd evaluation abnormal CP diagnosis with RT, of 8 patients, there was agreement in 2 (25.0%) patients.

**Table 4:** Agreement between cytopathological diagnosis abnormal or not and presence or absence of RT in the cells of the smear

		1st re-evaluation					
		PAP(−) RT^1^(−)	PAP(+) RT^1^(−)	PAP(−) RT^1^(+)	PAP(+) RT^1^(+)	Total	Kappa (95%CI)
Original	PAP(−) RT(−)	14	9	2	0	25	0.098 (0.001;0.22)
	PAP(+) RT(−)	9	11	2	3	25	
	PAP(−) RT(+)	17	1	3	0	21	
	PAP(+) RT(+)	6	1	14	3	24	
	Total	46	22	21	6	95	
2nd re-evaluation	PAP(−) RT(−)	12	3	2	1	18	0.281 (0.14;0.41)
	PAP(+) RT(−)	11	17	2	2	32	
	PAP(−) RT(+)	21	1	13	1	36	
	PAP(+) RT(+)	2	1	3	2	8	
	Total	46	22	20	6	94	

*PAP(−)* Normal Pap test, *PAP(+)* Abnormal Pap test, *RT(−)* No radiotherapy, *RT(+)* Radiotherapy, *1* Identification of post radiotherapy effects in cells

The length of time between the end of RT and the date of cytology abnormality averaged 848 days, with a standard deviation of 1278 days and a median of 364 days. No association between the cytological alterations and the median time between the end of RT and the cytological diagnosis was observed (Table 5).

**Table 5:** List of the cytological abnormalities according to median time after end of radiotherapy

Cytological Abnormalities	Time < 364 days	Time ≥ 364 days	*P*-value*
ASC-US and LSIL	3 (60.0%)	2 (40.0%)	1.00
AGC, HSIL and ASC-H	9 (47.4%)	10 (52.6%)	1.00

*Fisher’s exact test

*AGC* Atypical Glandular cells, *ASC-H* Atypical Squamous Cells cannot exclude HSIL, *ASC-US* Atypical Squamous Cells of Undetermined Significance, *HSIL* High Grade Squamous Intraepithelial lesion, *LSIL* Low Grade Squamous Intraepithelial lesion

## Discussion

The results suggest that radiotherapy has a strong influence in CP diagnosis also in normal or abnormal CP results. When we compared the interobserver agreement concerning CP diagnosis in patients that received RT, the agreement was only 14% and 12.5% in normal and abnormal CP diagnosis, respectively. This is one of the reasons that cytology is not recommended to use in follow-up of women treated with radiotherapy for cervical cancer [10].

In our study, the intraobserver evaluation (K^3^), with presence of RT, demonstrated worst diagnostic agreement related to a decreased values of kappa (unweighted K^3^ = 0.37) when compared to the same evaluation in all patients regardless RT status (weighted K^3^ = 0.52) and patients without RT (weighted K^3^ = 0.47). Also, Settakorn et al. [14] study, which compared interobserver agreement of two cytopathologists in cytologic interpretation of liquid based cytology preparations with conventional Papanicolaou smears, showed reasonable agreement (weighted K = 0.37, 95%CI = 0.31–0.44 and weighted K = 0.40, 95%CI = 0.33–0.46). In addition, the agreement about the presence of RT signals in the smear was low (44%). These findings corroborate that it is difficult to recognize the cytological RT signals in the cervical cells.

Regarding the interobserver agreement, Stein et al. [12] demonstrated that there was no difference in the measurement of agreement between the observations (Kappa values) according to the status of radiotherapy and CA in the interpretation among cytotechnologists in manual and automated screening. The results of this work evidenced few differences in weighted kappa values when compared presence of RT (K^1^ = 0.016 95%CI: 0.03–0.06 e K^2^ = 0.041 95%CI: 0.01–0.09) and absence of RT (K^1^ = 0.149 95%CI: 0.02–0.32 e K^2^ = 0.120 95%CI: 0.04–0.31) in interobserver agreement between CP diagnosis. Nevertheless, this range of values keep in the same category of slight agreement. In spite of the similar conditions of slides analysis, our study demonstrated that the presence of radiotherapy influences the morphological classifications defined in the cytological diagnosis when evaluated by the same observer, which differs from the study by Stein et al., who evaluated only interobserver agreement.

According to the review undertaken for this article, this is the first study to correlate intraobserver reproducibility of cytological diagnosis with RT information. This work demonstrated that this agreement of the cytological diagnosis between two analysis conducted by the same cytotechnologist was moderate (K = 0.52) regarding the limits of the first and second CP re-evaluation. In evaluations by Tsilalis et al. [15], regarding telecytological diagnosis of cervical smears not related to RT, the intraobserver variability was near perfect among the five cytopathologists and presented a gradual increase during the diagnostic evaluations with values for kappa ​​ranging from 0.76 to 1.00. In a Norwegian study, the number of Pap smears evaluated as abnormal (ASC-US+) by the four pathologists varied from 61 to 85. The number of high-grade cytology (ASC-H+) varied from 26 to 50. There was moderate agreement (weighted kappa 0.45–0.58) between the observers [10]. In the ATHENA study, there were considerable differences among the laboratories both in overall cytological abnormal rates, ranging from 3.8 to 9.9%, and in sensitivity of cytology to detect CIN grade 2 or worse (CIN2+), from 42.0 to 73.0% [11].

The hypothesis here is that some patients who undergo RT may experience exfoliation of the epithelium over time (regeneration) and thus the cells under post radiotherapy effect may disappear giving rise to new cells without changes. This could explain the fact that patients in this study who underwent RT had no cytological changes from the radiation. In addition, some patients may have received vaginal hormone therapy with estrogen that could influence the disappearance of post radiotherapy CA.

It is relevant to note that this research includes an original methodology whose correlation of intraobserver reproducibility with prior RT information had not been studied to date. Although Stein et al. [16] point out that radiation produces a bias that favors the cytological categorization of uncertainty; this study emphasizes the need for cytotechnologists and cytopathologists to be able to recognize properly any type of abnormalities, regardless of whether they know the radiotherapy history of a slide. This is an important issue in order to improve the quality of the cytological interpretation and consequently reduce the errors of cytopathological classification.

The main limitation of this work was that the sample was small for the application of certain statistical tests, making it necessary to adapt some categories. However, the literature brings some studies with even smaller samples such as Lee et al. [17] with 50 randomized smear slides, as well as studies such as Stein with 10,165 cases of cervical cytology analyzed [16]. This study was conducted with only two cytopathologists and it is therefore difficult to generalize the findings to a broader population of cytopathologists. This could be a limitation for interobserver agreement, wherever the focus is in intraobserver reproducibility.

The strengths of this study lie in its prospective characteristic, in the use of liquid-based cytology that makes it possible to obtain a better smear with less unsatisfactory results, as well as in the evaluation of the intraobserver reproducibility. In addition, it is definitely a strength that the pathologist in the pathology department of BCH routinely reassessed all the dubious cytological diagnoses.

The intraobserver agreement was only moderate, suggesting there may have been influence of the knowledge of previous RT in the interpretation of the slides by the cytotechnologist. It was expected that this knowledge would lead to better agreement in the second CP re-evaluation, but this was not observed. Therefore, it has been shown that reproducibility is best when done by the same observer, since they are able to better identify proposed diagnoses.

The interobserver and intraobserver analysis concerning CP results agreement had high discrepancy in patients that received RT. Adequate knowledge about these abnormalities is imperative in order to avoid false positive diagnoses, since most of them are not associated with new intraepithelial/invasive lesions or tumor recurrence. Cytology and HPV cotesting increases the sensitivity and may reduce false positive diagnoses [18].

## Conclusion

This study found that there is a difficulty on the part of the cytotechnologist to recognize cellular abnormalities due exclusively to RT when this information is not provided. It is important to emphasize, therefore, the importance of the personnel to inform at the time of the collection whether or not the patient received RT. The authors believe that cytology should not be indicated in follow-up after radiotherapy for cervical cancer treatment.

## Additional file

Additional file 1: Values for the cytological diagnoses according to RT Status. (DOCX 44 kb)

## Abbreviations

AGC: Atypical Glandular Cells
ASC-H: Atypical Squamous Cells is not possible to exclude High-grade Intraepithelial Lesion
ASC-US: Atypical Squamous Cells of Undetermined Significance
BCH: Barretos Cancer Hospital
CA: Cytological Abnormalities
CP: Cytopathology
HSIL: High Grade Squamous Intraepithelial Lesion
RT: Radiotherapy
