# Supplementary material for: Pelvic radiotherapy for cervical cancer affects importantly the reproducibility of cytological alterations evaluation
Source: BMC Clin Pathol. 2018 Oct 5;18:11. doi: 10.1186/s12907-018-0078-z (PMC6173841; doi:10.1186/s12907-018-0078-z)
Supplement: Supplementary file 1 — Values for the cytological diagnoses according to RT Status. (DOCX 44 kb) [file 12907_2018_78_MOESM1_ESM.docx]

**Supplementary Table 1. K^1^: Original vs First re-evaluation (all patients)**

|  |  |  |  | First CP |  |  |  |  |
| --- | --- | --- | --- | --- | --- | --- | --- | --- |
| Original CP | Negative | ASC-US | LSIL | AGC | ASC-H | HSIL | ADENO/CEC | Total |
| Negative | 36 | 2 | 3 | 0 | 5 | 0 | 0 | 46 |
| ASC-US | 5 | 3 | 1 | 0 | 0 | 0 | 0 | 9 |
| LSIL | 4 | 2 | 1 | 0 | 0 | 0 | 0 | 7 |
| AGC | 1 | 0 | 0 | 1 | 0 | 1 | 0 | 3 |
| ASC-H | 15 | 3 | 0 | 0 | 0 | 0 | 0 | 18 |
| HSIL | 6 | 3 | 0 | 0 | 0 | 0 | 0 | 9 |
| ADENO/CEC | 0 | 0 | 0 | 0 | 1 | 2 | 0 | 3 |
| Total | 67 | 13 | 5 | 1 | 6 | 3 | 0 | 95 |

ADENO/CEC: Adenocarcinoma; AGC: Atypical Glandular cells; ASC-H: Atypical Squamous Cells cannot exclude HSIL; ASC-US: Atypical Squamous Cells of Undetermined Significance; HSIL: High Grade Squamous Intraepithelial lesion; LSIL: Low Grade Squamous Intraepithelial lesion.

**Supplementary Table 2. K^2^: Original vs Second re-evaluation (all patients)**

|  |  |  |  | Second CP |  |  |  |  |
| --- | --- | --- | --- | --- | --- | --- | --- | --- |
| Original CP | Negative | ASC-US | LSIL | AGC | ASC-H | HSIL | AEDO/CEC | Total |
| Negative | 29 | 99 | 1 | 1 | 5 | 1 | 0 | 46 |
| ASC-US | 7 | 2 | 0 | 0 | 0 | 0 | 0 | 9 |
| LSIL | 3 | 2 | 2 | 0 | 0 | 0 | 0 | 7 |
| AGC | 1 | 0 | 0 | 1 | 0 | 1 | 0 | 3 |
| ASC-H | 10 | 6 | 0 | 0 | 1 | 0 | 0 | 17 |
| HSIL | 4 | 5 | 0 | 0 | 0 | 0 | 0 | 9 |
| ADENO/CEC | 0 | 1 | 0 | 0 | 0 | 2 | 0 | 3 |
| Total | 54 | 25 | 3 | 2 | 6 | 4 | 0 | 94 |

ADENO/CEC: Adenocarcinoma; AGC: Atypical Glandular cells; ASC-H: Atypical Squamous Cells cannot exclude HSIL; ASC-US: Atypical Squamous Cells of Undetermined Significance; HSIL: High Grade Squamous Intraepithelial lesion; LSIL: Low Grade Squamous Intraepithelial lesion

**Supplementary Table 3. K^3^: First re-evaluation vs Second re-evaluation (all patients)**

|  |  |  |  | Second CP |  |  |  |
| --- | --- | --- | --- | --- | --- | --- | --- |
| First CP | Negative | ASC-US | LSIL | AGC | ASC-H | HSIL | Total |
| Negative | 48 | 13 | 1 | 1 | 3 | 0 | 66 |
| ASC-US | 4 | 9 | 0 | 0 | 0 | 0 | 13 |
| LSIL | 2 | 1 | 2 | 0 | 0 | 0 | 5 |
| AGC | 0 | 0 | 0 | 1 | 0 | 0 | 1 |
| ASC-H | 0 | 2 | 0 | 0 | 3 | 1 | 6 |
| HSIL | 0 | 0 | 0 | 0 | 0 | 3 | 3 |
| Total | 54 | 25 | 3 | 2 | 6 | 4 | 94 |

**Supplementary Table 4. K^1^: Original vs First re-evaluation (patients not submitted to radiotherapy)**

|  |  |  |  | First CP |  |  |  |  |
| --- | --- | --- | --- | --- | --- | --- | --- | --- |
| Original CP | Negative | ASC-US | LSIL | AGC | ASC-H | HSIL | ADENO/CEC | Total |
| Negative | 16 | 1 | 3 | 0 | 5 | 0 | 0 | 25 |
| ASC-US | 3 | 3 | 1 | 0 | 0 | 0 | 0 | 7 |
| LSIL | 1 | 2 | 1 | 0 | 0 | 0 | 0 | 4 |
| AGC | 1 | 0 | 0 | 1 | 0 | 1 | 0 | 3 |
| ASC-H | 3 | 1 | 0 | 0 | 0 | 0 | 0 | 4 |
| HSIL | 3 | 1 | 0 | 0 | 0 | 0 | 0 | 4 |
| ADENO/CEC | 0 | 0 | 0 | 0 | 1 | 2 | 0 | 3 |
| Total | 27 | 8 | 5 | 1 | 6 | 3 | 0 | 50 |

ADENO/CEC: Adenocarcinoma; AGC: Atypical Glandular cells; ASC-H: Atypical Squamous Cells cannot exclude HSIL; ASC-US: Atypical Squamous Cells of Undetermined Significance; HSIL: High Grade Squamous Intraepithelial lesion; LSIL: Low Grade Squamous Intraepithelial lesion.

**Supplementary Table 5. K^2^: Original vs Second re-evaluation (patients not submitted to radiotherapy)**

|  |  |  |  | Second CP |  |  |  |  |
| --- | --- | --- | --- | --- | --- | --- | --- | --- |
| Original CP | Negative | ASC-US | LSIL | AGC | ASC-H | HSIL | AEDO/CEC | Total |
| Negative | 9 | 8 | 1 | 1 | 5 | 1 | 0 | 25 |
| ASC-US | 5 | 2 | 0 | 0 | 0 | 0 | 0 | 7 |
| LSIL | 0 | 2 | 2 | 0 | 0 | 0 | 0 | 4 |
| AGC | 1 | 0 | 0 | 1 | 0 | 1 | 0 | 3 |
| ASC-H | 1 | 2 | 0 | 0 | 1 | 0 | 0 | 4 |
| HSIL | 2 | 2 | 0 | 0 | 0 | 0 | 0 | 4 |
| ADENO/CEC | 0 | 1 | 0 | 0 | 0 | 2 | 0 | 3 |
| Total | 18 | 17 | 3 | 2 | 6 | 4 | 0 | 50 |

ADENO/CEC: Adenocarcinoma; AGC: Atypical Glandular cells; ASC-H: Atypical Squamous Cells cannot exclude HSIL; ASC-US: Atypical Squamous Cells of Undetermined Significance; HSIL: High Grade Squamous Intraepithelial lesion; LSIL: Low Grade Squamous Intraepithelial lesion.

**Supplementary Table 6. K^3^: First re-evaluation vs Second re-evaluation (patients not submitted to radiotherapy)**

|  |  |  |  | Second CP |  |  |  |
| --- | --- | --- | --- | --- | --- | --- | --- |
| First CP | Negative | ASC-US | LSIL | AGC | ASC-H | HSIL | Total |
| Negative | 14 | 8 | 1 | 1 | 3 | 0 | 27 |
| ASC-US | 2 | 6 | 0 | 0 | 0 | 0 | 8 |
| LSIL | 2 | 1 | 2 | 0 | 0 | 0 | 5 |
| AGC | 0 | 0 | 0 | 1 | 0 | 0 | 1 |
| ASC-H | 0 | 2 | 0 | 0 | 3 | 1 | 6 |
| HSIL | 0 | 0 | 0 | 0 | 0 | 3 | 3 |
| Total | 18 | 17 | 3 | 2 | 6 | 4 | 50 |

AGC: Atypical Glandular cells; ASC-H: Atypical Squamous Cells cannot exclude HSIL; ASC-US: Atypical Squamous Cells of Undetermined Significance; HSIL: High Grade Squamous Intraepithelial lesion; LSIL: Low Grade Squamous Intraepithelial lesion.

**Supplementary Table 7. K^1^: Original vs First re-evaluation (patients submitted to radiotherapy)**

|  |  |  | First CP |  |  |  |
| --- | --- | --- | --- | --- | --- | --- |
| Original CP | Negative | ASC-US | LSIL | ASC-H | HSIL | Total |
| Negative | 20 | 1 | 0 | 0 | 0 | 21 |
| ASC-US | 2 | 0 | 0 | 0 | 0 | 2 |
| LSIL | 3 | 0 | 0 | 0 | 0 | 3 |
| ASC-H | 12 | 2 | 0 | 0 | 0 | 14 |
| HSIL | 3 | 2 | 0 | 0 | 0 | 5 |
| Total | 50 | 5 | 0 | 0 | 0 | 45 |

ASC-H: Atypical Squamous Cells cannot exclude HSIL; ASC-US: Atypical Squamous Cells of Undetermined Significance; HSIL: High Grade Squamous Intraepithelial lesion; LSIL: Low Grade Squamous Intraepithelial lesion.

**Supplementary Table 8. K^2^: Original vs Second re-evaluation (patients submitted to radiotherapy)**

|  |  |  | Second CP |  |  |  |
| --- | --- | --- | --- | --- | --- | --- |
| Original CP | Negative | ASC-US | LSIL | ASC-H | HSIL | Total |
| Negative | 20 | 1 | 0 | 0 | 0 | 21 |
| ASC-US | 2 | 0 | 0 | 0 | 0 | 2 |
| LSIL | 3 | 0 | 0 | 0 | 0 | 3 |
| ASC-H | 9 | 4 | 0 | 0 | 0 | 13 |
| HSIL | 2 | 3 | 0 | 0 | 0 | 5 |
| Total | 36 | 8 | 0 | 0 | 0 | 44 |

ASC-H: Atypical Squamous Cells cannot exclude HSIL; ASC-US: Atypical Squamous Cells of Undetermined Significance; HSIL: High Grade Squamous Intraepithelial lesion; LSIL: Low Grade Squamous Intraepithelial lesion.

**Supplementary Table 9. K^3^: First re-evaluation vs Second re-evaluation (patients submitted to radiotherapy)**

|  | Second CP |  |  |
| --- | --- | --- | --- |
| First CP | Negative | ASC-US | Total |
| Negative | 34 | 5 | 39 |
| ASC-US | 2 | 3 | 5 |
| Total | 36 | 0 | 44 |

ASC-US: Atypical Squamous Cells of Undetermined Significance.
